# Unravelling the Data Retention Mechanisms under Thermal Stress on 2D Memristors

**DOI:** 10.1021/acsomega.3c03200

**Published:** 2023-07-20

**Authors:** Samuel Aldana, Hongzhou Zhang

**Affiliations:** † Centre for Research on Adaptive Nanostructures and Nanodevices (CRANN) and Advanced Materials and Bioengineering Research (AMBER) Research Centers, Trinity College Dublin, Dublin D02 PN40, Ireland; ‡ School of Physics, Trinity College Dublin, Dublin D02 PN40, Ireland

## Abstract

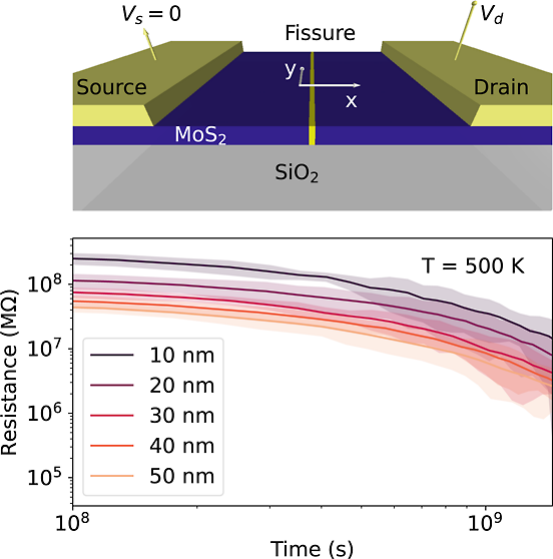

Memristors based on two-dimensional (2D) materials are a rapidly growing research area due to their potential in energy-efficient in-memory processing and neuromorphic computing. However, the data retention of these emerging memristors remains sparsely investigated, despite its crucial importance to device performance and reliability. In this study, we employ kinetic Monte–Carlo simulations to investigate the data retention of a 2D planar memristor. The operation of the memristor depends on field-driven on defect migration, while thermal diffusion gradually evens the defect distribution, leading to the degradation of the high resistance state (HRS) and diminishing the ON/OFF ratio. Notably, we examine the resilience of devices based on single crystals of transition metal dichalcogenides (TMDs) in harsh environments. Specifically, our simulations show that MoS_2_-based devices have negligible degradation after 10 years of thermal annealing at 400 K. Furthermore, the variability in data retention lifetime across different temperatures is less than 22%, indicating a relatively consistent performance over a range of thermal conditions. We also demonstrate that device miniaturization does not compromise data retention lifetime. Moreover, employing materials with higher activation energy for defect migration can significantly enhance data retention at the cost of increased switching voltage. These findings shed light on the behavior of 2D memristors and pave the way for their optimization in practical applications.

## Introduction

Memristors are gaining relevance as emerging electronic devices during the last decades because of their remarkable potential applications in diverse fields, such as multibit non-volatile memories,^1^ encryption,^2^ and neuromorphic computing.^3−5^ Memristors outperform flash technology, the current dominant memory, in scalability (down to ∼2 nm),^6^ energy-efficiency (∼10 fJ),^7^ switching speed (85 ps),^8^ endurance (>10^12^ cycles),^9^ and data retention (projections over 10 years at 85 °C).^10^ However, these devices must meet a range of reliability requirements before they can be used in large integrated circuits.^11^ For example, stringent requirements on device reliability are necessary in electronics operating under extreme conditions, like nuclear reactors, aerospace industries, or automotive factories where circuits are exposed to radiation.^12^ Therefore, developing resilient and reliable memristors capable of withstanding both radiation and high temperatures (>125 °C) would be beneficial to various applications.^13^ Furthermore, device overuse or exposure to electrical or thermal stress can cause device wear-out and failure.^14^

The information stored in a memristor is represented by its resistance value. Thus, any unintended changes in the resistance value can lead to data loss and it is necessary to establish a tolerance level of acceptable resistance change, which may differ according to the specific application.^14^ The time taken for the resistance to change beyond this tolerance level is called the data retention lifetime. To ensure that the data retention of a memristor stays within the tolerance range, the stability of low resistance states (LRS), and high resistance states (HRS) must be monitored over time. To achieve long-term data retention in memristors often requires higher voltages or longer exposition times to the electric stress to operate the device. This phenomenon is known as the time–voltage dilemma.^15,16^ Data retention in memristors is usually challenged by aggressive environments, such as high temperature, humidity, or radiation exposure. The data loss in non-filamentary devices is expected to be progressive, often manifesting after prolonged stress. Under such aggressive conditions, the migration of defects within the device’s functional layers is accelerated, resulting in random migrations, and disrupting the initially well-organized resistance states.^17^ Consequently, exposure to aggressive environments not only affects data retention lifetime but also impairs device operation, leading to the degradation of resistance ratios.^18^ Moreover, since the basic operation of memristors involves a limited number of ion migrations, there can be variabilities in the movement of those ions, which can lead to differences in device resistance.^19^ The LRS in conductive filament (CF)-based devices is determined by the presence of a percolation path between the electrodes and the data retention is the time this CF takes to vanish under ambient conditions,^14^ leading the device to the HRS. Since the data retention depends on the CF stability, it also depends on the compliance current used for the forming or SET process that determines the CF size.^20,21^ Previous studies have reported promising data retention results for vertical memristors based on two-dimensional (2D) materials, such as MoS_2_, with data retention lifetimes up to 10^5^ s at ambient temperature.^22−25^

To date, there is a lack of studies exploring long-term data retention of planar memristors under various temperatures. The knowledge of memristors at elevated temperatures provides a practical route to assess its reliability at their normal working conditions. Conducting 10 year long experiments to measure their long-term data retention is impractical, and therefore accelerated testing methods are widely used to estimate device failure and data loss. One common method involves exposing devices to temperatures above 100 °C for extended periods of time, typically ranging from several hours up to a few hundred hours, which accelerates the aging process of memristors. Researchers can then utilize the Arrhenius equation^14,26−28^ to extrapolate device performance. On the other hand, simulations offer a cost-effective way to estimate long-term performance and help to improve the device design, reliability, endurance, and data retention lifetime.^29^

Improving the performance and longevity of memristive devices requires a thorough understanding of their failure mechanisms and data retention limitations. This knowledge is essential for developing new materials and architectures that can enhance the reliability and efficiency of these devices. While in situ techniques, electrical characterization, and structural analysis are useful tools to characterize memristive devices with respect to their failure mechanisms and data retention capacity, they have certain limitations due to experimental conditions or system complexity. Hence, integrating theoretical models with computational simulations offer valuable insights into atomic-scale processes that underlie device behavior responsible for data retention failures. Besides, simulations allow for the exploration of a wide range of experimental conditions and device architectures, as well as the ability to isolate and study specific physical processes that may be difficult to observe experimentally.^30^ For instance, ab initio calculations can provide accurate results for the activation energy that controls the resistive switching process or the resistance state degradation.^31,32^ However, reproducing the specific degradation process that leads to device failure may be challenging, as this process can occur in a period beyond the scope of these techniques. On the other hand, compact models can be calibrated to reproduce the average behavior of the device.^33−36^ These models can reproduce the general process of degradation,^37^ while they may lack a description of the microscopic processes of the system, such as the random thermal-triggered migration of the particles that leads to device failure. The kinetic Monte–Carlo (kMC) algorithm is a well-established technique that facilitates the comprehension of stochastic phenomena, such as temperature-induced random migration of defects.^29^ Its flexibility enables thorough analysis of thermodynamic and kinetic behavior of fundamental transitions occurring even at complex spatial and temporal scales. Moreover, owing to its stochastic nature, kMC can effectively replicate atomic diffusion or chemical reactions, establishing a link between the microscopic configuration and macroscopic system behavior.^38^ Researchers have utilized kMC to study stochastic phenomena such as resistive switching processes, cycle-to-cycle variability, and retention time tests under thermal stress in memristors.^29,39−45^ The kMC approach offers atomic-scale details and can access regimes beyond the reach of other techniques, such as molecular dynamics. This is because the diffusion process can be expressed on time and length scales much larger than those associated with atomic vibrations.^38,46^ By conducting kMC simulations, we can estimate how temperature affects various physical processes, including atom migration, which significantly influences the data retention lifetime of memristive devices. Hence, kMC simulations offer a valuable tool for evaluating the device’s stability over time at different temperatures, determining the temperature range where the device can maintain its desired performance, and assessing the consistency of data retention at a specific temperature.

The present study focuses on a unique forming-free planar memristor based on 2D materials. It is well established that the electrical stress of the forming process causes uncontrollable material modification,^47−49^ leading to device variabilities,^49,50^ and hindering device testing.^51^ Reliable forming of individual cells in large-scale memory chips is impractical due to economic constraints.^52^ Therefore, forming-free memristors provide significant technological advantages. However, it is yet to be investigated if forming-free devices can offer benefits in terms of data retention. Moreover, 2D memristors exhibit significant potential for energy-efficient neuromorphic computations.^4,53^ The planar architecture enables effective gate tuning and multi-terminal operation, improving device controllability and enabling complex neural functionalities.^4,54^ Intriguingly, the inherent low capacitance of these planar memristors facilitates fast switching and contributes to overall low power consumption. We have utilized a physical simulator based on the kMC algorithm to investigate the retention time of this planar device when subjected to high thermal stress. Our research focuses on exploring the failure process and degradation in the performance of these devices, as well as examining their temperature tolerance and how factors such as device size and activation energy for defect migration impact retention time. Through our investigation, we also analyze how thermal stress affects data retention consistency and consider the influence of defect distribution on retention time. Our study does not aim to provide precise and accurate results, but rather to offer valuable information about the general dynamics and trends related to the retention time of 2D planar devices under high thermal stress. These key findings provide insights into how to prevent device failure and improve the retention time in the technology by modulating the migration energy and distribution of defects in the channel.

## Simulation Description

**Figure 1:**
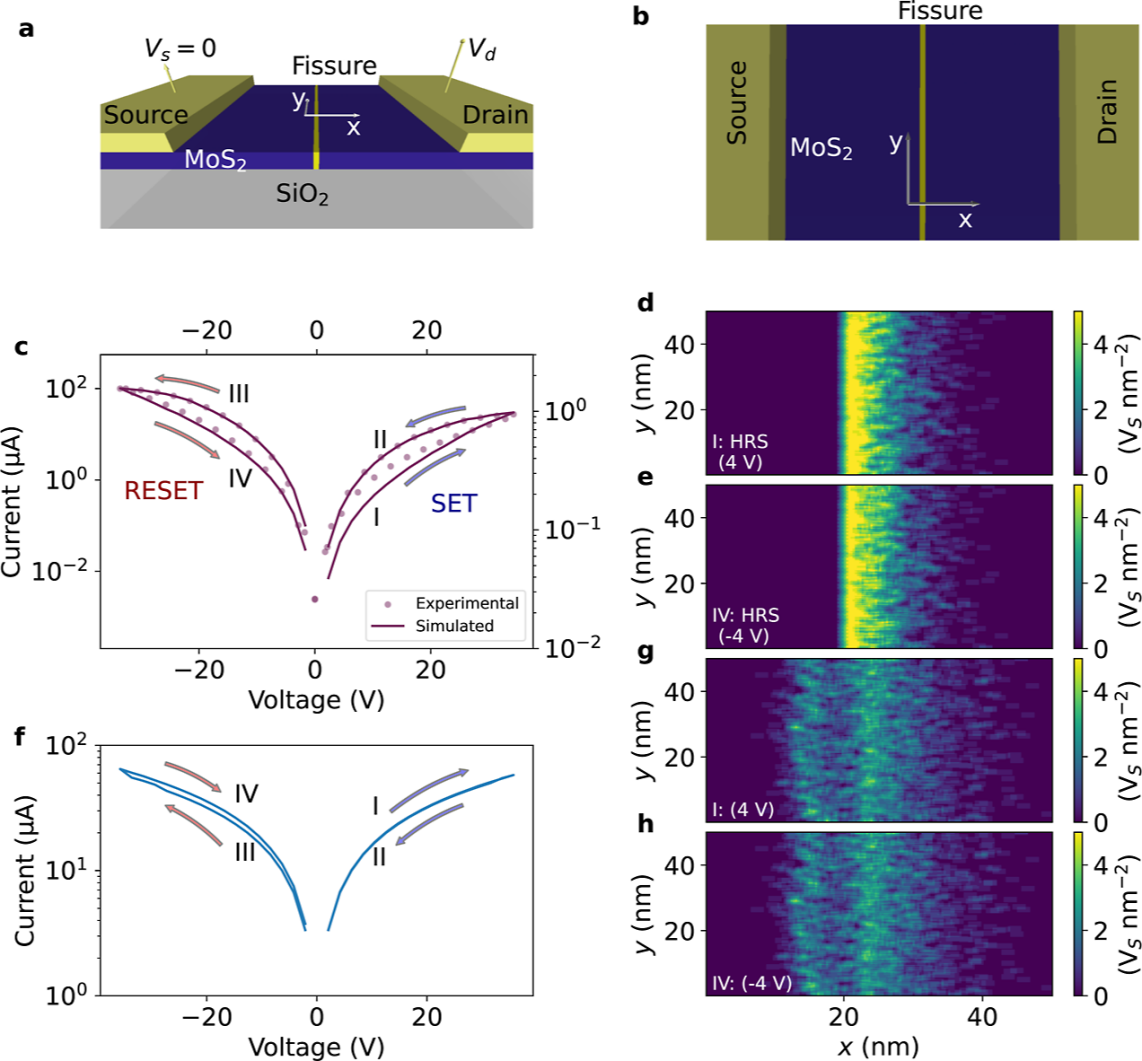
Device failure. (a) Schematic illustration of the device structure highlighting the fissure of defects in the MoS_2_ channel (yellow line) over a SiO_2_ substrate, with the source electrode grounded. (b) Top view of the schematic device structure, with the *x*-axis-oriented perpendicular to the electrodes and the *y*-axis-aligned parallel to them. (c) *I*–*V* hysteresis loop with the experimental data plotted against the bottom and left axes, while simulated data are plotted against the top and right axes. For the simulation, the initial defect profile of the device exhibits a skewed Gaussian distribution with a peak density of 5.64 V_s_·nm^–2^ and 68% of defects within 8 nm width at ambient temperature (300 K). Voltage sweeps between 35 and −35 V at a voltage ramp rate of 2.1 V/s. Representative states of the switching process are marked with numerals on the loop. The SET process occurs from state I to state II, while the RESET process occurs from state III to state IV. The local defect density at the state I and IV (HRS) during the voltage sweeps in (c) are (d,e), respectively. (f) simulated data for a device with identical conditions as in (c), after thermal annealing for 7 days at 600 K. Numerals on the loop follow the same criteria as in (c). The local defect density at the states I and IV during the voltage sweeps in (f) are (g,h), respectively.

The present study employs a rejection-free kMC physical simulator, the variant with all events successful.^55^ The kMC simulator accurately reproduces the defect migration in 2D materials and computes the device’s resistance by considering the microscopic defect configuration in the channel, using an empirical relationship.^56^ The simulator has been previously employed to investigate 2D planar-type devices with a spatially varying defect concentration induced by ion irradiation in the channel, which enables the resistive switching effect.^57^ The ion irradiation was conducted using a helium ion beam microscope, which selectively removes sulfur atoms from the MoS_2_.^56^ The investigated device (see the device schematic and the top view in Figure 1a,b), based on 2H–MoS_2_, does not require a forming process and exhibits a non-filamentary RS mechanism, where the migration of defects under an applied electric field modulates the device’s resistance.^58^ The device fabrication involved the use of mechanically exfoliated monolayer, ensuring the absence of grain boundaries, while the predominant defects resulting from this fabrication method are single sulfur vacancies.^59^ Furthermore, it is worth noting that MoS_2_ is susceptible to the surrounding environment. However, given that our experimental devices were tested within a vacuum chamber with a base pressure of ∼10^–5^ mbar, we did not incorporate the interaction with the surrounding environment into our simulations. See more details about the fabrication and characterization processes in Supporting Information, Section S1. In this study, we explored the retention of the HRS and the device operation under varying temperatures by monitoring the microscopic evolution of the system.

The emergence of the HRS is attributed to the accumulation of defects in the fissure region (see the yellow region in Figure 1d,e). The local defect density  determines the local resistance  via the empirical relationship , where *n* is a fitting parameter that can be determined from experimental data.^56^ To calculate the local defect density, we average over 6 × 6 grid points (3.2 nm^2^) at each grid point. The stochastic thermal migration of defects leads to an isotropic spreading in the channel that leads to a reduction in device resistance. Defect migration is a thermally activated process characterized by transition rates that are parameterized by temperature, activation energy, and the vibration constant of particles. The process follows the Arrhenius equation: ,^38^ where κ_B_ is the Boltzmann constant, ν = 7 × 10^13^ s^–1^ the vibration constant, *T* the temperature, and *E*_A_ the activation energy of defect migration (2.297 eV for MoS_2_).^4^ Typically, transition metal dichalcogenides have relatively high activation energy for defect migration, which ranges from 2.1 to 2.9 eV.^4,60^ The generation of defects in MoS_2_ is negligible due to the high energy barriers for defect generation, for example, 5.85 eV for sulfur vacancies^61^ and >5 eV antisite defects.^62^ In 2D MoS_2,_ defect clustering tends to form rows. Since the energy barrier for disrupting these rows is significantly lower than the energy barrier for defect migration,^61^ the timescales associated with cluster disruption and defect migration differ significantly. Therefore, the simulation disregards defect clustering due to its negligible impact on the data retention lifetime. We note that elevated temperatures can lead to material and interface degradation, which were not considered in the simulation framework. For example, the sublimation point of MoS_2_ is 723 K, which is in proximity to the maximum temperature used in the study (650 K). Consequently, caution must be exercised when extrapolating the obtained results to experimental conditions.

The transition rates are organized in a balanced binary tree and a binary search method is used to find the chosen event through the kMC technique.^55^ The time step follows the relationship: , where rand is a uniform random number between 0 and 1 and ∑Γ is the summation of the transition rates for all possible events. The simulation domain is 50 × 50 nm and the asymmetric distribution of defects follows a skewed Gaussian distribution.

The simulation incorporates the electric field description by solving the Poisson equation , using the finite difference method to account for particle interactions. In this equation, φ(*x*,*y*) represents the electric potential corresponding to the charge distribution ρ_c_(*x*,*y*) in a MoS_2_ monolayer with a relative permittivity ε = 3.2.^63^ Diritchlet boundary conditions are applied at the electrodes, while Neumann boundary conditions are implemented for the lateral faces. The activation energy is then modulated by the local electric field as follows: *E*_A_ = *E*_A_^0^ – ***b***·***F***(*x*,*y*),^42^ where ***F***(*x*,*y*) is the electric field, *b* = *p*_0_·[(2 + ε)/3] is the polarization factor, *p*_0_ = 15 e·nm^64^ and *E*_A_^0^ are the zero-field effective activation energy.

## Results and Discussion

Figure 1 illustrates the device schematic and the thermal degradation of the device operation, highlighting its implications on resistive switching. The simulation starts with a skewed Gaussian defect distribution at ambient temperature (300 K), with a peak density of 5.64 V_s_·nm^–2^ and 68% of defects within 8 nm width. The device exhibits typical resistive switching evidenced by the quasi-static pinched hysteresis loops in Figure 1c. The local defect density at the initial stage (Figure 1d) and after completing the resistive switching process (Figure 1e) at 4 and −4 V, respectively, are revealed in the colormaps. In the HRS, defects accumulate in a narrow region, that is, the fissure region, which leads to a large resistance. The SET process occurs by applying a positive external electric field that drives the defects away from the fissure region (from state I to II), which reduces the peak density of the defects and hence the resistance. Conversely, during the RESET process, a negative electric field moves defects toward the fissure (from states III to IV), recovering the initial defect configuration and hence the resistance state. The reconfiguration of the defects results in a resistance ratio of 1.6. The observed discrepancies in current magnitudes between experimental and simulated data in Figure 1c stem from the different device sizes along the *y*-axis (see Figure 1b). Specifically, the experimental device has a width of 29.4 μm, while the simulation domain is 50 nm. Due to the non-filamentary nature of the conduction mechanism, the total resistance of the device scales with the device width.^57^

Figure 1f exhibits an *I*–*V* simulated curve for a device with identical conditions as in Figure 1c, but after thermal annealing for 7 days at 600 K. After the annealing, the resistance drops almost 2 orders of magnitude and resistive switching disappears upon applying identical quasi-static electrical stimuli, as shown in Figure 1f. Figure S1 in the Supporting Information presents the different stages of the local defect density in the channel during the annealing process. Furthermore, Figure S2 exhibits the corresponding microscopic configurations at each stage, complementing the information presented in Figure S1. The positive electric field has a negligible effect on the device resistance (from states I to II), whereas applying a negative electric field fails to recover the pre-annealing accumulation of defects (from states III to IV), leading to a reduction in resistance (>11%) instead of an increase. Thermal stress during the annealing process induces defects to migrate from high-density to low-density areas, which in turn leads to lower electric fields.^57^ This device failure is attributed to the low electric field, which renders the device incapable of regaining the accumulation of defects shown in Figure 1d or 1e due to infrequent field-driven migrations (Figure S3 in Supporting Information). Different stages of the resistive switching process (I to IV) are in the Supporting Information (Figure S4).

**Figure 2:**
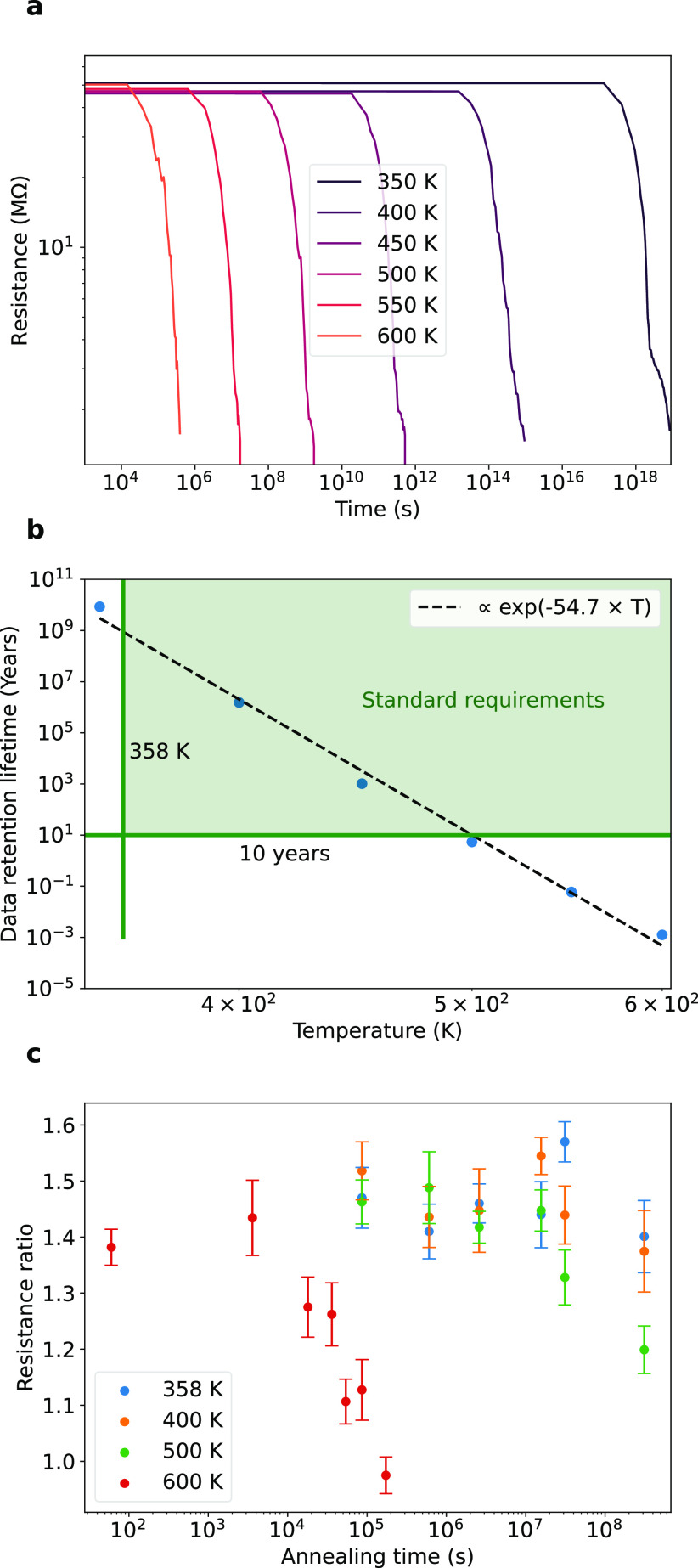
Thermal degradation of an individual device. (a) Temporal evolution of resistance under thermal stress (between 350 and 600 K). The activation energy for defect migration is *E*_A_ = 2.297 eV (corresponding to MoS_2_).^4^ The simulations start from a skewed Gaussian distribution of defects, using ρ = 5.64 V_s_·nm^–2^ and a width of 8 nm. (b) Data retention lifetime with the temperature assuming a data loss occurs when the resistance decreases by 20%. The green area represents the data retention lifetime surpassing the standard requirements. (c) Resistance ratio as a function of annealing time at four different temperatures (358, 400, 500, and 600 K). The resistance ratio is calculated by averaging 10 simulated RS cycles, performed at room temperature after the annealing time indicated on the *x*-axis.

Figure 2 describes the data retention lifetime and the degradation of the resistance ratio in relation to temperature for an individual device. All the simulation starts with a skewed Gaussian defect distribution, with a peak density of 5.64 V_s_·nm^–2^ and 68% of defects within 8 nm width. Figure 2a exhibits the temporal evolution of resistance at various thermal stresses. As shown, the degradation of the HRS expedites by several orders of magnitude with increasing temperature. At the highest temperature (600 K) examined, the device’s resistance deteriorates over a period of days, while at a lower temperature (450 K), it takes thousands of years to see a similar degree of degradation. Given that memristors store information as resistance values, it is crucial to establish the permissible range of resistance change to maintain data integrity. Balancing the tolerance level with the storage capacity is a key part of the device’s performance. A higher tolerance level reduces the risk of data loss but limits the range of intermediate resistance states, resulting in lower storage capacity. Conversely, a lower tolerance level allows more intermediate resistance states, but it reduces the data retention lifetime. Consequently, the choice of tolerance depends on the specific application and the desired data retention. For our study, we selected a maximum resistance change of 20% before concluding that the data are lost. Figure 2b depicts the data retention lifetime using the curves in Figure 2a assuming the previous data loss criterion ( > 20%). The data retention time follows the typical Arrhenius law. The green-shaded area in Figure 2b corresponds to a data retention lifetime surpassing the standard requirement of 10 years at 358 K, thus establishing the device as a suitable candidate for deployment in challenging environments. Figure 2c elucidates the influence of different annealing processes on device operation, specifically focusing on the device’s resistance ratio. The systems are exposed to thermal stress at various durations, utilizing four different temperatures: 358, 400, 500, and 600 K. Subsequently, the systems are cooled back to ambient temperature (300 K) before performing the device switching process. The resistance ratio (*y*-axis) is determined by averaging 10 simulated RS cycles with voltage sweeps ranging from 35 to −35 V and a voltage ramp rate of 2.1 V/s. The annealing process at 358 and 400 K has a negligible effect on the device resistance ratio for up to 10 years. However, subjecting the device to thermal stress of 500 K over a decade result in a remarkable degradation in the device’s resistance ratio, with a drop of about 20%. Further, some days of exposure to temperatures of 600 K adversely impact the device’s performance, with its switching capability significantly compromised.

**Figure 3:**
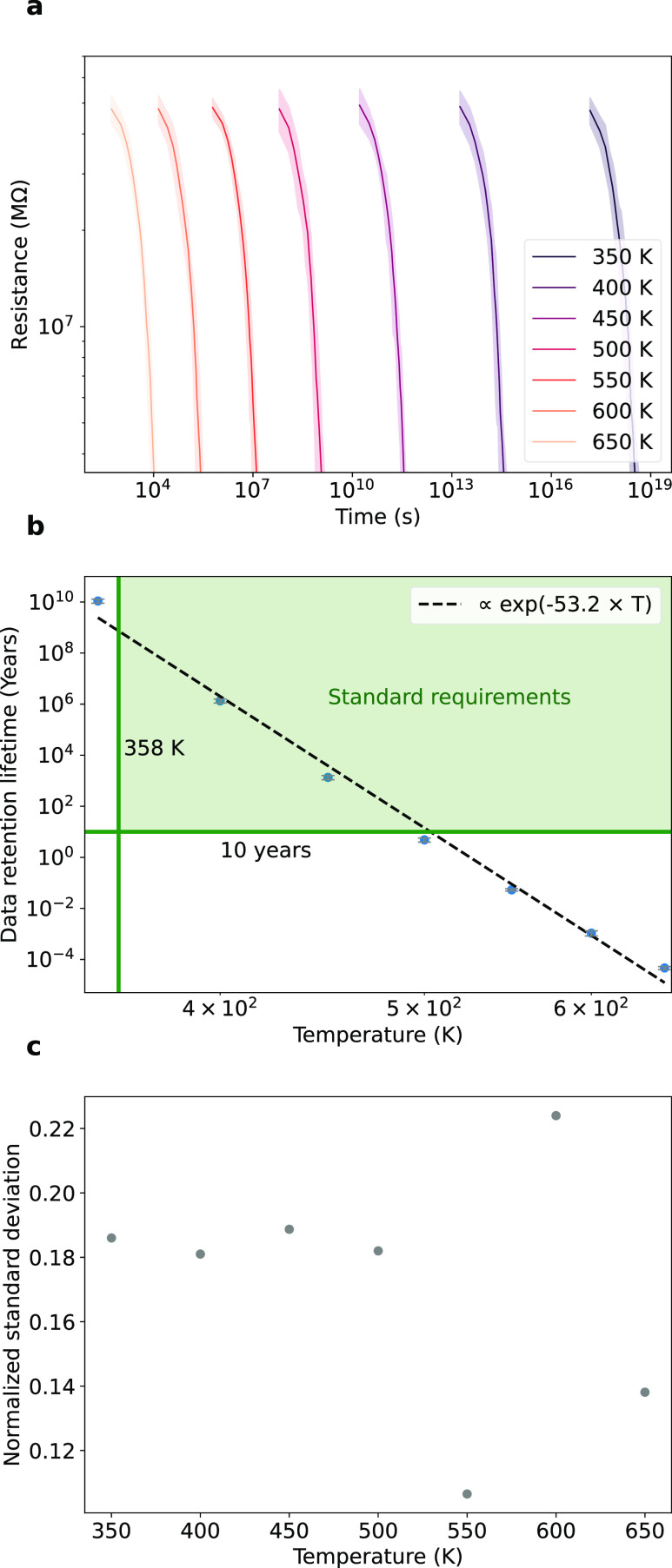
Data retention lifetime variability between devices. (a) Temporal evolution of the resistance under thermal stresses (from 350 to 650 K) with the same initial parameters: start from a skewed Gaussian distribution of defects, ρ = 5.64 V_s_·nm^–2^, width of 8 nm and activation energy *E*_A_ = 2.297 eV. Ten simulations per color with the solid line, the mean and the shaded region, the 95% prediction interval. (b) Data retention lifetime with the temperature assuming a data loss occurs when the resistance decreases by 20%. The green area represents the data retention lifetime surpassing the standard requirements. Error bars represent the standard deviation. (c) Shows the normalized standard deviation (the standard deviation divided by the mean) to compare the variability at different temperatures.

Variations in data retention can significantly impact practical applications, potentially leading to unreliable performance, shortened data retention lifetimes, data corruption, and loss of accuracy. Figure 3 explores the impact of temperature on data retention variability across multiple devices. Figure 3a presents the variations in the degradation of resistance at temperatures ranging from 350 to 650 K. We conducted 10 simulations, each representing a different device. Although the simulations for each temperature share the same parameters, they possess distinct initial microscopic vacancy configurations due to the random introduction of defects. The solid line is the mean resistance, while the shaded region indicates the 95% prediction interval. The temperature speeds up the deterioration of the resistance, but its influence on the observed variations among the different simulations is limited. This is clear from the narrow-shaded region for each temperature. To quantify the extent of variability in data retention lifetime induced by temperature, we present the mean values with error bars representing the standard deviation in Figure 3b. The green-shaded area in Figure 3b corresponds to a data retention lifetime surpassing the standard requirement. To compare the variability at different temperatures, Figure 3c presents the dependence of the normalized standard deviation (calculated by dividing the standard deviation by the mean) on temperature. It can be observed that the standard deviation of data retention lifetime is less than 22% of the mean value. This range of variability for different temperatures offers a guide to help predict and control the behavior of the device, which should be considered in the design and implementation of practical applications, such as memory or neuromorphic computing.

**Figure 4:**
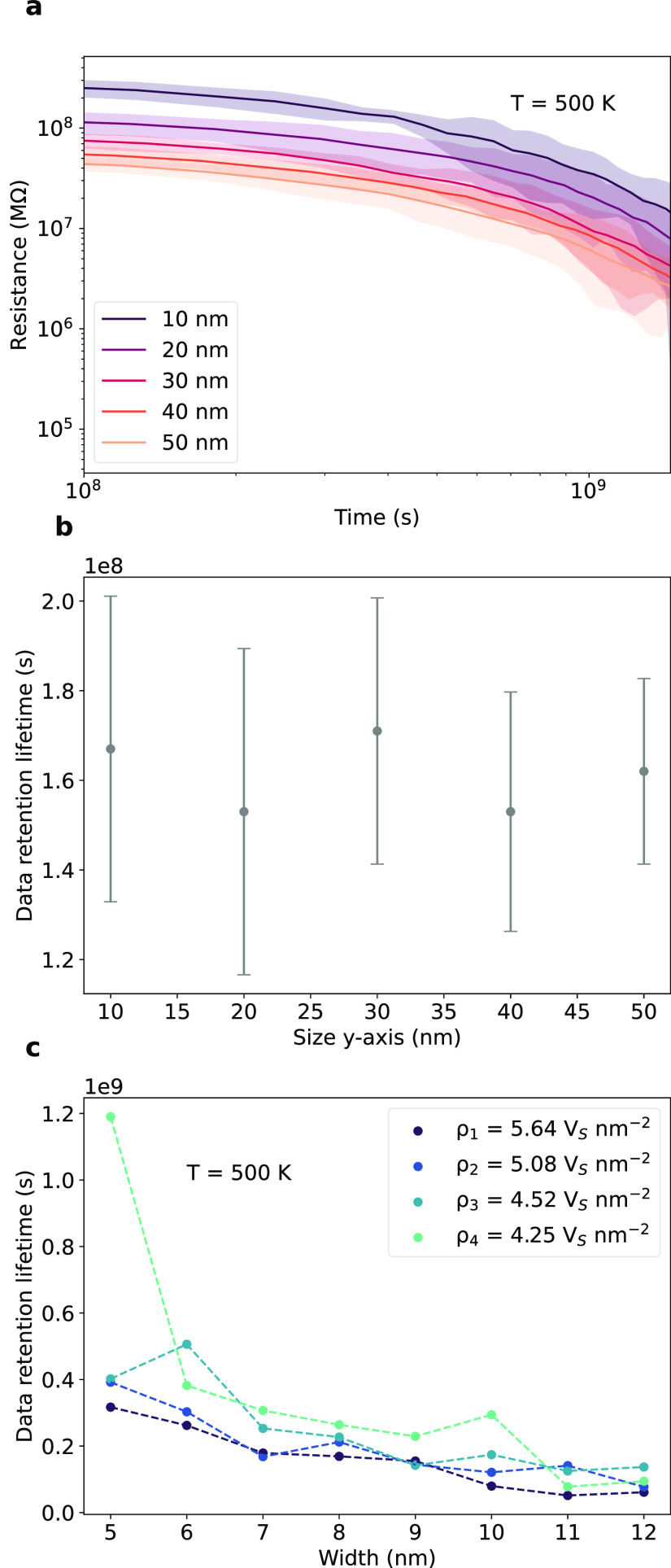
Data retention lifetime with device scalability. (a) Temporal evolution of resistance under thermal stress (500 K) for devices of varying *y*-axis sizes (10, 20, 30, 40, and 50 nm) with a fixed x-size (50 nm) and fissure region width (8 nm). Ten simulations were conducted, with the solid line the mean and the shaded region the 95% prediction interval. (b) Data retention lifetime as a function of device size (*y*-axis). Data retention is averaged over 10 simulations and the error bars denote standard deviation. Data loss occurs when the resistance decreases by 20%. (c) Data retention lifetime under thermal stress (500 K) for different fissure region widths (regions with 68% of defects) and densities (ρ_1_ = 5.64 V_s_·nm^–2^, ρ_2_ = 5.08 V_s_·nm^–2^, ρ_3_ = 4.52 V_s_·nm^–2^, ρ_4_ = 4.25 V_s_·nm^–2^). Lighter blue curves correspond to lower density. All simulations start with a skewed Gaussian distribution of defects and activation energy of defect migration of 2.297 eV.

The device size has a key impact on some device electrical features such as the resistance.^57^Figure 4 focuses on the effect of device size on the data retention lifetime. While the data retention of filamentary-based devices is linked to the cell size, which can affect the stability of the filament,^21^ our device rely on a distributed resistive switching mechanism.^57,58^ This results in a different relationship between device size and its electrical properties. Specifically, in Figure 4a, we investigate the impact of the dimension parallel to the electrodes (*y*-axis) on the temporal evolution of resistance, where a broader-shaded area indicates greater resistance variations among devices. Although smaller sizes exhibit higher resistance, data retention lifetime does not exhibit a clear correlation with device dimension, as shown in Figure 4b. Additionally, reducing the fissure region width and defect density slightly increases data retention time, as demonstrated in Figure 4c. Our findings suggest that the miniaturization of devices does not significantly compromise data retention lifetime. However, it is worth noting that the device miniaturization does have an impact on resistance ratio and variability, making the device less suitable for certain applications.^57^ These results provide valuable guidance for tailoring memristors with specific features and trade-offs for different applications.

**Figure 5:**
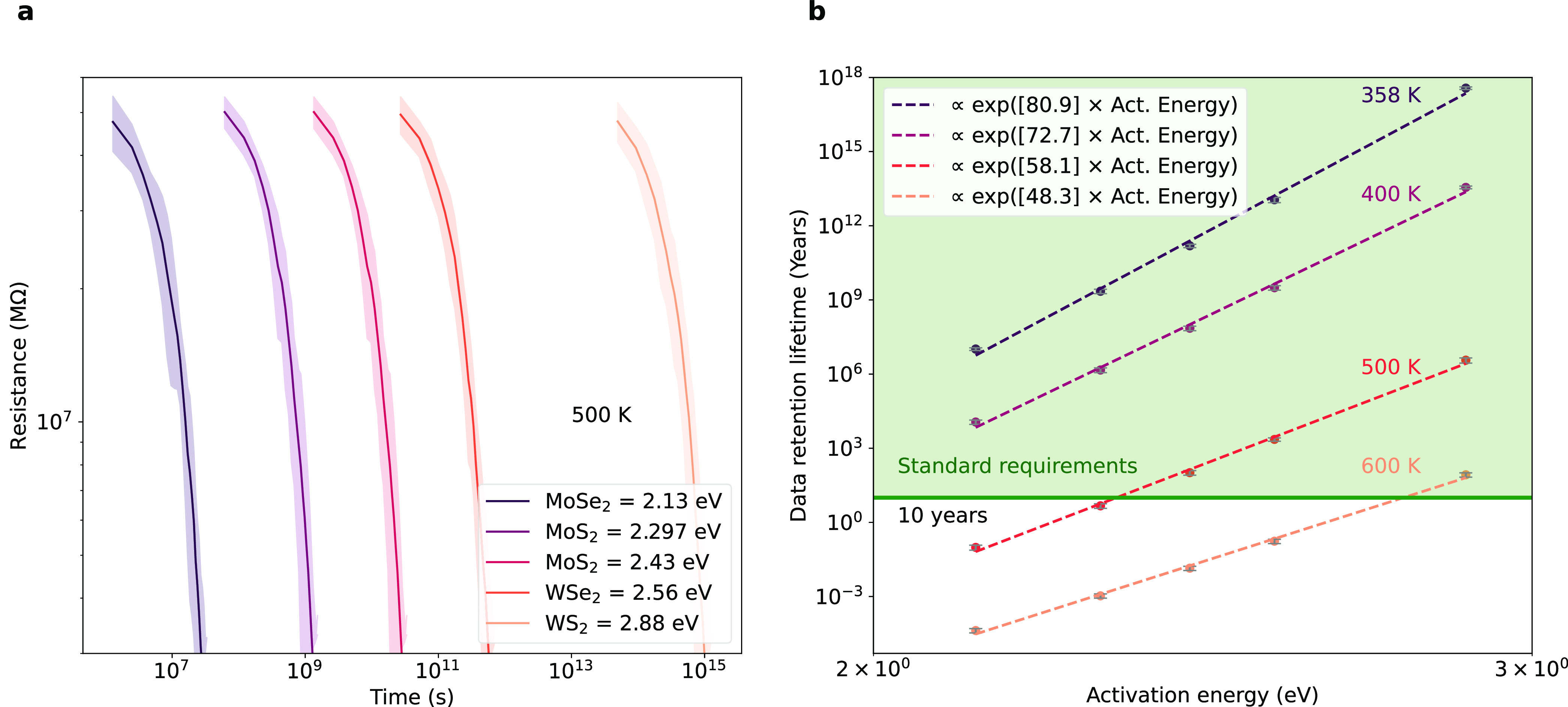
Influence of activation energy on data retention lifetime. The simulations start with a skewed Gaussian defect distribution with a peak density of 5.64 V_s_·nm^–2^ and 68% of defects within 8 nm width. (a) Temporal evolution of resistance under thermal stress (500 K) for different activation energies of defect migration with typical values for transition metal dichalcogenides: MoSe_2_ (2.13 eV^60^), MoS_2_ (2.297 eV^4^ and 2.43 eV^60^), WSe_2_ (2.56 eV^60^), and WS_2_ (2.88 eV^60^). Ten simulations are conducted for each activation energy and the shaded region indicates the 95% prediction interval. (b) data retention lifetime as a function of defect migration activation energy for varying temperature. Data loss occurs when the resistance decreases by 20%. The green area represents the data retention lifetime surpassing the standard requirements. Each point is the mean of 10 simulations and error bars represent the standard deviation.

In Figure 5, we investigate the impact of activation energies on device data retention lifetime. As shown in Figure 5a, systems with higher activation energy for defect diffusion require longer exposure to thermal stress to achieve the same resistance reduction in the device. Figure 5b reveals that data retention lifetime can improve from 40 days at 500 K for the case of 2.13 eV (MoSe_2_) to millions of years for the case of 2.88 eV (WS_2_). At temperatures below 400 K, all the activation energies considered result in data retention lifetimes exceeding 10 years. However, at 600 K, only the case with an activation energy of 2.88 eV achieves a data retention lifetime of over 10 years. It is important to recognize that higher activation energies correspond to higher switching voltages required to operate the device, making the switching process more energy-intensive^52^ and leading to the time-voltage dilemma.^15^ It is also noteworthy that many filament-based memristors rely on migrating cations or vacancies with migration energies typically below 0.3 eV for cations^52,65^ and above 1 eV for vacancies.^66^ In general, these energies are far from the typical values for TMDs, suggesting that shorter data retention lifetimes can be expected in such materials. Furthermore, longer data retention times and increased resilience to harsh environments enable lower tolerance levels without compromising the integrity of the stored data and resulting in a greater number of possible intermediate resistance states and hence higher storage capacity.

## Conclusions

We employed kMC simulations and gained a comprehensive understanding of the thermal degradation process in the 2D planar memristor. Our results reveal the collapse of the HRS and the deterioration of the resistance ratio under thermal stress produced by the thermal dispersion of defect accumulation. Importantly, our findings have implications for experimental studies, highlighting several key points: (1) Devices based on single crystal of TMDs exhibit remarkable resilience in harsh environments. For example, our simulations demonstrate that MoS_2_-based devices experience negligible degradation in device operation and resistance ratio after 10 years of thermal annealing below 400 K. (2) The variability in data retention lifetime across different temperatures is less than 22%, indicating a relatively consistent performance over a range of thermal conditions. (3) Enhancing data retention lifetime and resilience to harsh environments can be achieved by employing materials with higher activation energies for defect migration. However, it is essential to consider that this improvement may come at the expense of increased switching voltage. (4) Longer data retention times and increased resilience enable lower tolerance levels without compromising the integrity of the stored data. This, in turn, allows for an increased number of intermediate resistance states, thereby augmenting the overall storage capacity. (5) The miniaturization of the device can improve data retention lifetime, but it also leads to a deterioration in the resistance ratio. Our findings provide valuable insights for designing and implementing memristors for energy-efficient in-memory processing and neuromorphic computing.

## Supporting Information Available

Details of the thermal diffusion of defects in the channel, device failure, and the microscopic configuration at different stages of the resistive switching process (PDF)
