# Supplementary material for: Unravelling the Data Retention Mechanisms under Thermal Stress on 2D Memristors
Source: ACS Omega. 2023 Jul 20;8(30):27543–52. doi: 10.1021/acsomega.3c03200 (PMC10398860; doi:10.1021/acsomega.3c03200)
Supplement: Supplementary file 1 — ao3c03200_si_001.pdf [file ao3c03200_si_001.pdf]

Supplementary Information: Unravelling the Data Retention Mechanisms under Thermal Stress on 2D Memristors

*Samuel Aldana** *and Hongzhou Zhang*

Section 1: Device fabrication and characterization

Single crystals of MoS_2 were mechanically exfoliated from bulk sources (SPI Supplies) using adhesive tape. The design of electrode patterns was accomplished using Raith Nanopatterning software, and electron beam lithography (FEI Strata DB235 SEM) was employed for their fabrication. A PMMA A9 resist was utilized at a dose of 340 $\mu C \cdot cm^{-2}$, followed by a 30-second development in MIBK/IPA (1:3) solution. Metallization involved the use of an electron beam evaporator (Detech DE400) to deposit Ti/Au (10/80 nm) pads over a large area, followed by lift-off in warm acetone.

The milling patterns were designed using the NanoPatterning Visualization Engine software. The patterns involved a single-direction pixel-wide scan, precisely traced in a single sweep without any retracing. To induce memristive switching, a delivered ion linear dose of approximately 1.6 $pC \cdot \mu m^{-1}$ was employed with a Helium Ion Microscope. This dose was achieved with a beam current of 1.5 pA and an aperture of 10 μm. The probe size was evaluated to be less than 3 nm and was determined using the GaussFit module in ImageJ software.

The experimental setup involved testing the samples within the vacuum chamber of a customized scanning electron microscope (Zeiss EVO), operating at a base pressure of approximately 10^{-5} mbar. To establish contact with the EBL-deposited pads serving as source and drain terminals, Imina miBot piezoelectric tungsten probes were employed. The data acquisition process was performed using a semiconductor analyzer (Agilent B2912A) interfaced with Keysight software.

For more comprehensive information regarding the fabrication process of the device, electrical characterization, as well as SEM, TEM, Raman, and Photoluminescence characterization, please refer to our previous study.[1]

Section 2: Thermal dispersion of defects in the channel

Figure S1a exhibits the evolution of the defect density profile under high thermal stress of 600 K for more than four days, ultimately leading to device failure. The accumulation of defects, which determines the HRS, is dispersed by thermal stress (see Figure S1a, the five density profiles at different times from t = 0 s to t = $3.8 \cdot 10^5$ s). Consequently, there is a constant decrease in resistance. The corresponding local defect density at each time is given in Figure S1b to S1f, while the microscopic configuration is in Figure S2a to S2f.

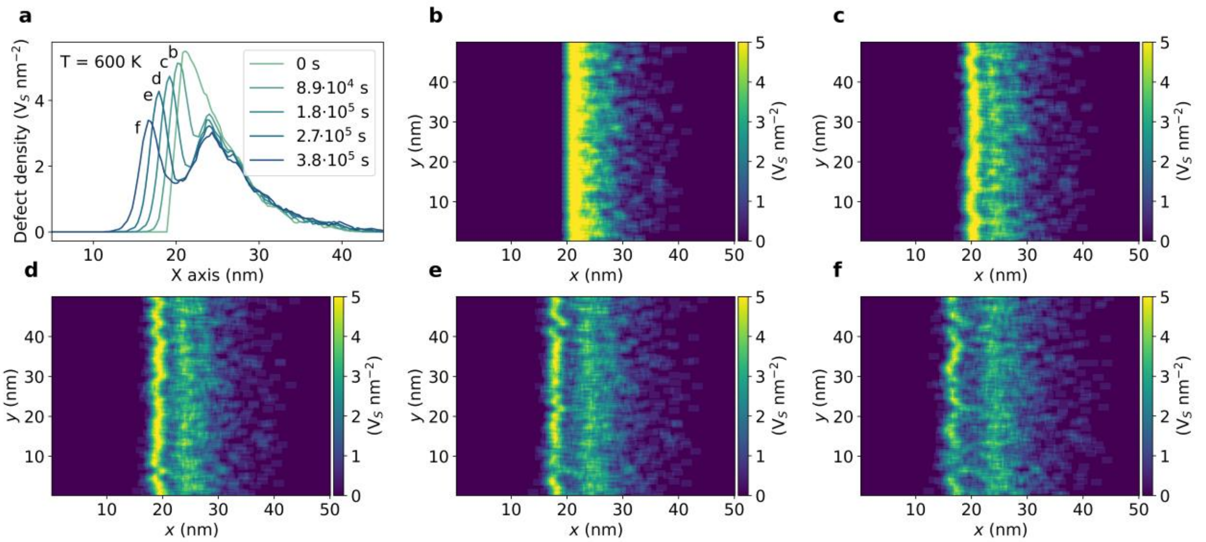

Figure S1: Thermal diffusion of defects. Local defect density in the device channel under thermal stress (600 K). The activation energy for defect migration is $E_A = 2.297\, eV$ (corresponding to MoS2).[2] The simulations start from a skewed Gaussian distribution of defects, using $\rho = 5.64\,\mathrm{Vs} \cdot \mathrm{nm}^2$ and a width of 8 nm. a) shows the defect density profile along the x-axis at 5 different stages ($t_1 = 0\, s,\ t_2 = 8.9 \times 10^4\, s,\ t_3 = 1.8 \times 10^5\, s,\ t_4 = 2.7 \times 10^5\, s$ and $t_5 = 3.8 \times 10^5\, s$). The lightest blue curve corresponds to t_1 and darker blues to the onward ones. b-f) are the local defect densities in the channel for the different times used in a), that is, b) is t_1, c) is t_2, d) is t_3, e) is t_4 and

f) is t_5.

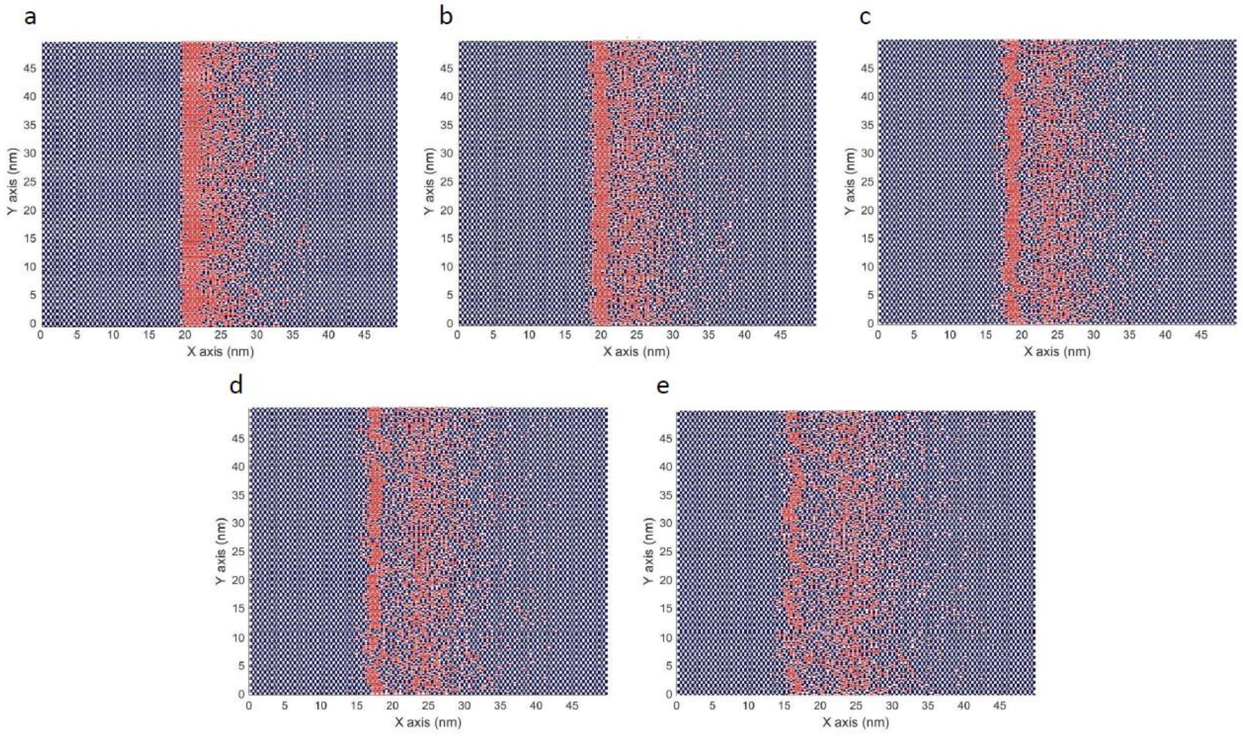

Figure S2: Microscopic configuration of the thermal diffusion of defects. The simulation is the same used in Figure S1 and show the microscopic configuration at the same 5 stages: a) ($t_1 = 0\ s$), b) $t_2 = 8.9 \times 10^4\ s$, c) $t_3 = 1.8 \times 10^5\ s$, d) $t_4 = 2.7 \times 10^5\ s$ and e) $t_5 = 3.8 \times 10^5\ s$. Red particles are sulfur vacancies in the MoS_2 monolayer.

Section 3: Device failure

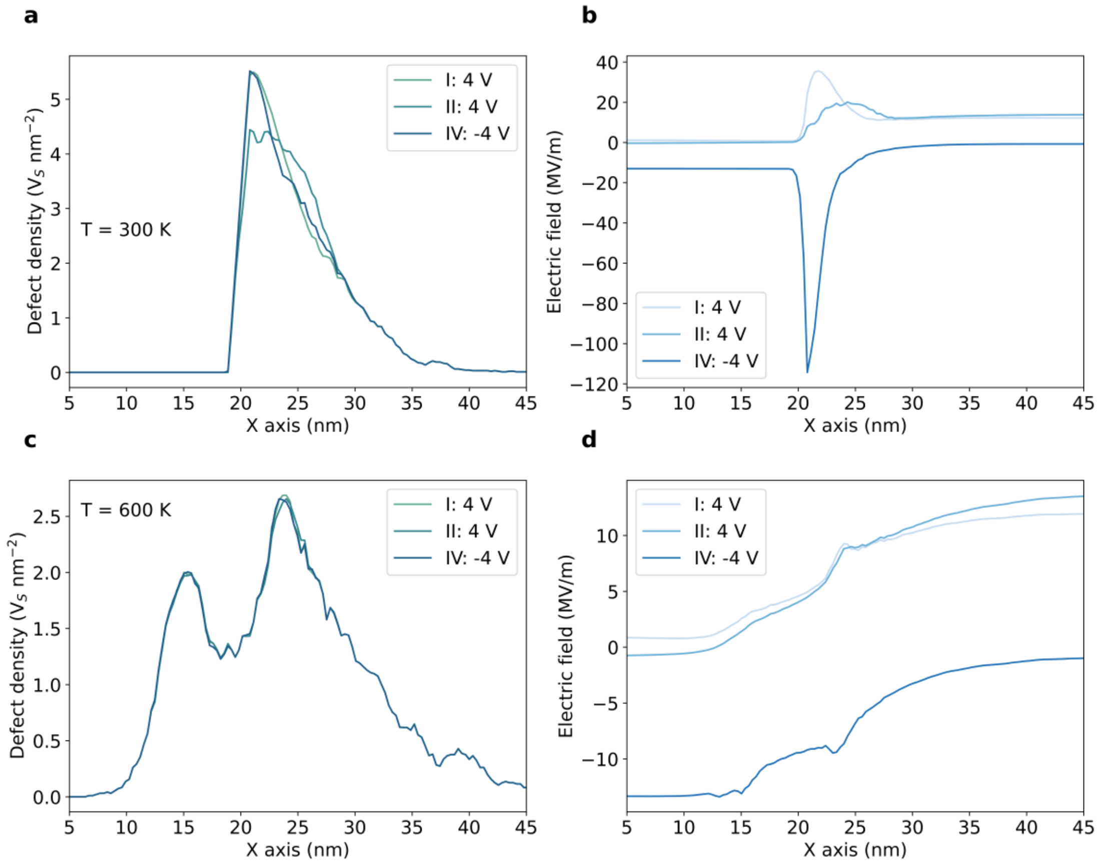

Figure S3: Device failure. Density and electric field profile in the x-axis corresponding to the simulations show in Figure 1. The initial defect profile of the device exhibits a skewed Gaussian distribution with a peak density of 5.64 $V_s \cdot nm^{-2}$ and 68% of defects within 8 nm width at ambient temperature (300 K). Voltage sweeps between 35 V and -35 V at a voltage ramp rate of 2.1 V/s. a) Density and b) electric field profile for a device operating in ambient conditions. c) Density and d) electric field profile for a device after thermal annealing for 7 days at 600 K. The profiles correspond to points I, II, and IV shown in Figure 1.

Figure S3 depicts the variations in the density and electric field profiles along the x-axis for two devices with similar conditions, except that one underwent thermal annealing at 600 K for seven days. The density of defects with spatial variation regulates the potential distribution in the channel when an external voltage is applied.[3] The thermal diffusion of defects in the channel results in a low electric field, which hinders the device from recovering the original accumulation of defects due to infrequent field-driven migration. Figure S3a and S3c show the density profiles at three distinct voltage sweep points corresponding to the same labels used in Figure 1 (I, II and IV). The electric field profiles at these points are displayed in Figure S3b and S3d.

Section 4: Local defect densities at different stages of the resistive switching process

Figure S4 show the microscopic configuration of two devices undergoing a voltage sweep between 35 V and -35 V, with a voltage ramp rate of 2.1 V/s, corresponding to points I to IV in Figure 1. We present the results for a device operating under ambient conditions and a device subjected to a seven-day annealing process at 600 K.

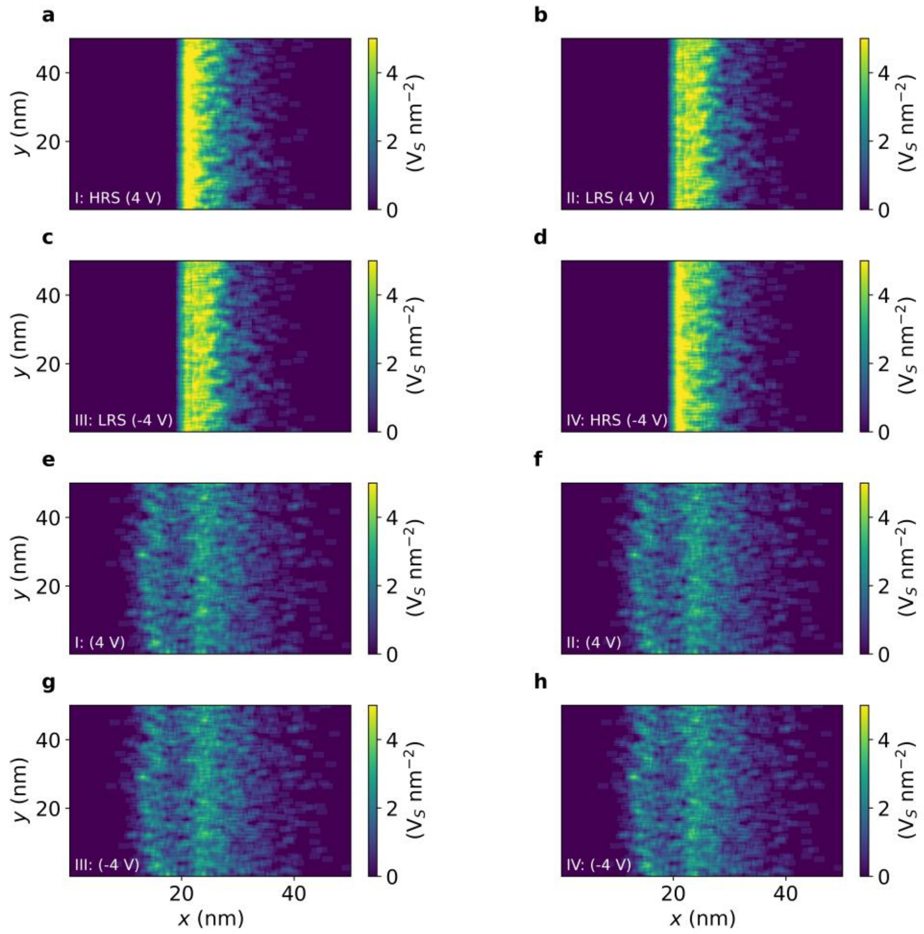

Figure S4: Local defect densities under voltage sweep. Different stages of the resistive switching cycle for a device without annealing (a - d) and after annealing (e - h). The labels I – IV correspond to the points shown in Figure 1a and 1b.

REFERENCES

(1) Jadwiszczak, J.; Keane, D.; Maguire, P.; Cullen, C. P.; Zhou, Y. B.; Song, H. D.; Downing, C.; Fox, D.; McEvoy, N.; Zhu, R.; et al. MoS2 Memtransistors Fabricated by Localized Helium Ion Beam Irradiation. *ACS Nano* **2019**, *13* (12), 14262-14273, Article. DOI: 10.1021/acsnano.9b07421.
(2) Wang, L.; Liao, W. G.; Wong, E. E. H.; Yu, Z. G.; Li, S. F.; Lim, Y. E. F.; Feng, X. W.; Tan, E. E. C.; Huang, X.; Chen, L.; et al. Artificial Synapses Based on Multiterminal Memtransistors for Neuromorphic Application. *Adv. Funct. Mater.* **2019**, *29* (25), 10, Article. DOI: 10.1002/adfm.201901106.
(3) Aldana, S.; Jadwiszczak, J.; Zhang, H. On the switching mechanism and optimisation of ion irradiation enabled 2D MoS2 memristors. *Nanoscale* **2023**. DOI: 10.1039/d2nr06810a.
